# Practical Remarks on the Two Most Prevailing Species of Cramp in the Stomach

**Published:** 1799-03

**Authors:** D. G. C. Conradi

**Affiliations:** Resident Physician at Northeim, in Germany


### *Practical Remarks on the two most prevailing Species of Cramp in the Stomach, by* D. G. C. Conradi, *M. D. Resident Physician at Northeim, in Germany.*

THE first species of spasm in the stomach originates from extreme debility, relaxation, and atony in that organ, whatever may have produced it as the primary or predisposing cause. The method of curing this distemper consists in the proper administration of alteratives and tonics, particularly those of the bitter kind; in adhering to a regular diet, and lastly in the use of chalybeates, after the necessary evacuations have been premised. This species of cramp is not so common as the next, but it merits notice here on this account chiefly, that the following may be the better illustrated by the comparison, according to the old adage: " *Opposita juxta se posita magis elucescunt.*"

The second species of spasmodic affections of the stomach arises from increased irritability and sensibility of the nerves connected with this important organ.—Briefly, every thing here exhibits a different appearance from what is observed in the former species, so that the first may not improperly be called the asthenic or passive cramp, and the second the sthenic or active. A good appetite and prompt digestion, a red and florid complexion, a strong and robust constitution, an irritable, active, and at the same time rigid fibre, are the characteristic symptoms of this second class. The affection is not violent in the beginning, but a pressure, stricture, and griping, rather than an acute pain, is felt in the region of the stomach. The patient has an oppressive sensation, as if something, not unlike a nail, were fixed behind the stomach: if the attack increase in violence, he complains of stitches in the breast and towards the back, and endeavours to procure relief by shifting his posture. The principal paroxysms are observed to take place generally in the afternoon, in consequeuce of bodily exercise immediately after dinner, the use of acid food and drink—and particularly after giving way to gusts of passion, such as terror, anger, grief, and anxiety.—Nothing is better calculated to prevent the approach of this disorder, than a wholesome and easily digestible meal, which with the moderate use of good old wine, will greatly contribute even towards effecting a cure, after it has become habitual.

This affection is often contracted or induced by persons subject to passionate emotions, on their neglecting to take an emetic occasionally, it is not, in general, attended with acidity, but rather, and most frequently is produced by a bilious acrimony; and it at length almost invariably degenerates into a nervous habit. This ſpecies of the diſeaſe is found to prevail moſt among the lower claſſes of people, whilſt that ariſing from atony, or relaxation, is more common among the opulent and epicures.

In its firſt attacks, and before it become habitual, the cramp may be conſiderably relieved by emetics, which frequently evacuate bile, together with an acid and corroſive matter. The following remedies, moreover, generally produce a decided effect in checking its actual progreſs: 1. Chalybeate waters, and eſpecially that of Pyrmont *, which has been obſerved in a number of caſes to afford inſtantaneous relief; 2. the *valeriana ſylveſtris*, the leaves of which, when reduced to powder, ſhould be taken three times a day, in doſes from two to four ſcruples, in cold water; the powder however muſt not be kept longer than a few days; this remedy is preferable even to the chalybeates; 3. the frequent drinking of cold water, and partial cold bathing of the region about the ſtomach, by which that preternatural ſenſibility in the digeſtive organs, ſo apt to generate the ſpaſm, is effectually extinguiſhed; and 4. in the ſlighter caſes of the diſeaſe, pills compounded of aſſafœtida and the extract of the valerian, will be found generally ſufficient to remove the complaint.

If the diſorder has become inveterate and perhaps incurable, palliatives will be the only remedies we can reſort to; and of theſe, opium and caſtor are the moſt efficacious: they are, indeed, not unfrequently ſuppoſed to be of eminent ſervice, even in ſcirrhoſities of the abdomen.

Dr. Conradi concludes with an injunction after the adminiſtration of emetics, in the firſt ſtages of this complaint, to avoid all bodily exerciſe, as this has a tendency to produce a return of the cramp.—Magneſia will be likewiſe of ſervice here, but neutral ſalts are not adviſeable; hence the plan of evacuating humours is generally productive of miſchief.—The remedies which on trial are found beneficial, ſhould not be too ſuddenly, but gradually relinquiſhed.—In complicated caſes of debility, our attention ſhould be directed to the tonic and ſtrengthening plan. The calx of Biſmuth, the Doctor obſerves, has never proved ſucceſsful, even in the largeſt doſes; although this powerful ſubſtance has been lately extolled by ſeveral practitioners as an almoſt infallible ſpecific.—With reſpect to diet, he adviſes to avoid wine and ſtrong beer between meals; long faſting, honey, gingerbread, ſweetmeats, and all kinds of paſtry are alſo obviouſly pernicious.

Inſtead

* An artificial Pyrmont-water may be prepared by boiling three ounces of iron-filings, and ſix ounces of cream of tartar, in a caſt-iron pot, with eight pints of water for about four hours, ſo as to reduce it to one half of its quantity; after which it ſhould be filtered through linen or cotton, and preſerved for uſe in bottles cloſely ſtopped. W.

Instead of beer at table, perſons ſubject to ſpaſms from debilitating cauſes, ſhould drink diſtilled water, and where this cannot be conveniently procured, they may ſubſtitute river-water previouſly boiled, and allowed to ſtand till it be perfectly cold.—Laſtly, to the long liſt of ſubſtances detrimental to the health of patients afflicted with the cramp, we may add the following articles of diet, ham, bacon, *fat* of pork and mutton, but above all, the habitual and immoderate uſe of *hot and ſtrong tea*, which is one of the moſt powerful cauſes prediſpoſing to that affection.

